# Proteomic, Fatty Acid and Mineral Profiles of PDO Arouquesa and Commercial Crossbred Beefs: A Tool for Certification

**DOI:** 10.3390/ani16010005

**Published:** 2025-12-19

**Authors:** Laura Sacarrão-Birrento, Sarah Schlosser, Karin Hummel, Ebrahim Razzazi-Fazeli, Cátia F. Martins, Miguel P. Mourato, José A. Silva, Severiano R. Silva, Susana P. Alves, Carlos A. Venâncio, Ingrid Miller, André M. de Almeida

**Affiliations:** 1 LEAF—Linking Landscape, Environment, Agriculture and Food Research Center, Instituto Superior de Agronomia, University of Lisbon, Tapada da Ajuda, 1349-017 Lisboa, Portugal; laurasvbirrento@isa.ulisboa.pt (L.S.-B.); catiamartins@isa.ulisboa.pt (C.F.M.); mmourato@isa.ulisboa.pt (M.P.M.); 2 Vetcore Facility for Research, University of Veterinary Medicine Vienna, Veterinaerplatz 1, 1210 Vienna, Austria; sarah.schlosser@vetmeduni.ac.at (S.S.);; 3 Associate Laboratory TERRA, Instituto Superior de Agronomia, University of Lisbon, Tapada da Ajuda, 1349-017 Lisboa, Portugal; 4 Veterinary and Animal Research Centre (CECAV), Associate Laboratory for Animal and Veterinary Sciences (AL4AnimalS), University of Trás-os-Montes e Alto Douro, 5000-801 Vila Real, Portugal; jasilva@utad.pt (J.A.S.); ssilva@utad.pt (S.R.S.); cvenanci@utad.pt (C.A.V.); 5 Animal Science Department, School of Agrarian and Veterinary Sciences (ECAV), University of Trás-os-Montes e Alto Douro, 5000-801 Vila Real, Portugal; 6 Centre for Interdisciplinary Research in Animal Health (CIISA), Associate Laboratory for Animal and Veterinary Sciences (AL4AnimalS), Faculty of Veterinary Medicine, University of Lisbon, Avenida da Universidade Técnica, 1300-477 Lisboa, Portugal; susanaalves@fmv.ulisboa.pt; 7 Centre for the Research and Technology of Agro-Environmental and Biological Sciences (CITAB), University of Trás-os-Montes and Alto Douro (UTAD), 5000-801 Vila Real, Portugal; 8 Centre of Biological Sciences, Department of Biological Sciences and Pathobiology, University of Veterinary Medicine Vienna, Veterinaerplatz 1, 1210 Vienna, Austria; ingrid.miller@vetmeduni.ac.at

**Keywords:** Arouquesa beef, PDO products, commercial beef, 2D-DIGE, meat quality

## Abstract

Protected Designation of Origin (PDO) beef products are recognized for their higher quality, although the native breeds used often show lower productivity compared to commercial crosses. This study aimed to compare the proteome profiles of Arouquesa and crossbred beef using 2D-DIGE, and evaluating crude protein, energy, and fatty acid composition. Arouquesa beef had higher energy values (*p* < 0.05), whereas crossbred samples showed greater protein content (*p* < 0.05). Moreover, Arouquesa beef had higher proportions of microminerals, namely zinc, iron, and manganese (*p* < 0.05). The Arouquesa breed also presented higher proportions of most fatty acids, including saturated, monounsaturated, and polyunsaturated. Proteomic analysis identified 34 differentially abundant proteoforms from 23 proteins. In Arouquesa, proteins associated with muscle contraction (MYLPF, TNNT1), glycolysis/gluconeogenesis (TPI1, GPD1), oxygen transport (HBB, HBA), and heat shock response (CRYAB, HSPB1) were more abundant or had altered spot patterns, suggesting their roles in meat tenderness, color, and fat-related characteristics. Conversely, crossbred beef showed elevated levels of glycolytic enzymes (PGM1, ENO3, and some proteoforms of TPI1 and GPI) and structural proteins (MYL1), related with higher muscle growth and different fiber composition. The identified proteins provide possible molecular markers that distinguish Arouquesa from commercial breeds. These validated proteins in the future can support breed characterization and verification of PDO certification specifications.

## Simple Summary

Beef certified under the Protected Designation of Origin (PDO) label, such as Arouquesa beef, is valued for its unique taste and quality. This is due to the traditional farming methods that are based on slow growth rates and the use of local breeds. However, these native breeds produce less meat than commercial crossbred animals. This study compared meat from Arouquesa cattle with meat from crossbred animals to understand the differences and to add value to PDO products. We measured basic nutritional values, including protein and energy content, and analyzed the fatty acid profile and the proteome. Arouquesa beef contained more energy and a healthier fatty acid profile. The proteome analysis revealed specific molecules that influence flavor, tenderness, and color in Arouquesa beef. These findings describe the molecular background of the distinctive qualities of traditional-produced meat. The identified proteins can also serve as possible biomarkers to authenticate PDO products, ensuring the specifications of high-quality beef linked to its traditional system. This study contributes by adding value to local breeds and traditional livestock systems that assists with sustainable agriculture and rural development.

## 1. Introduction

With the increase in world population and consequently higher demand for food products, beef represents a rich source of nutrients essential to human health such as protein, essential amino acids, iron, zinc, and several vitamins. Today, consumers are highly concerned about the quality and origin of meat products as well as their sustainability. The exotic sire breeds, such as Charolais and Limousin, are known for high growth rates and high-quality carcass, with a low percentage of fat and high muscle growth. For this reason, crossbreeding is used to improve the production of breeds with lower productivity, and some studies showed that, in fact, when local breeds are crossbred with exotic breeds, most meat productivity parameters, such as animals’ liveweights, carcass growth and yield, and dressing percentage, significantly improve [1,2]. However, beef from autochthonous breeds is considered a high-quality product as it has a distinctive flavor and texture due to the production systems based on natural grazing and a more gradual growth process [3]. In addition, these breeds have a robust resilience to challenging environmental conditions specific to their areas of origin. This adaptive capacity allows them to thrive in harsh local environments where other breeds might struggle, ensuring consistent performance and productivity [4]. Their use also allows higher efficiency in the use of resources as these breeds can use marginal areas that are not usable for crop production [5]. For these reasons, local breeds are often associated with more sustainable production practices, and consumers are more interested in these products, mainly if they are certified [6].

Arouquesa is an autochthonous Portuguese cattle breed produced in a traditional mountain system from where a well-known product originates—the Arouquesa PDO (Protected Designation of Origin) beef [7]. This is a product with specific flavor and aroma due to the production system [4], and such products are gaining more attention since they are associated with sustainability and high-quality organoleptic properties [8]. However, decreasing production rates and income in rural mountain regions are causing a general decrease of agricultural activities and rural exodus [4]. For this reason, it is of the utmost importance to improve production and increase the knowledge about these valuable genetic resources in order to assure high-quality products for the consumers, while at the same time making the public aware about their sustainable production systems. This will contribute to the value of local products, ultimately leading to local development in rural mountain communities.

Proteomics is widely used to study several aspects involved in the process of muscle-to-meat conversion and meat quality, such as tenderness, color, and pH [9,10,11,12,13]. These tools provide extensive knowledge about the metabolic pathways that can explain phenotypic responses to the production system [14]. Protein separation by 1- and 2-DE has been widely used in proteomics. However, an innovative technique such as 2D-DIGE that employs a pre-electrophoretic labeling process, using minimal amounts of fluorescent dyes to tag proteins, allows the analysis of multiple samples on a single gel [15]. 2D-DIGE has been applied in several studies related to meat quality to investigate factors that influence for instance tenderness [16,17], differences between different types of muscles [18], or muscles from animals subjected to different feeding regimens [19]. Additionally, studies concerning proteomics of muscle from different breeds have also been conducted. Chaze and co-workers [20] studied the muscle proteome using 2-DE of three French breeds (Blonde d’Aquitaine, Limousin, and Charolais) and showed different potential biomarkers of tenderness such as alpha actin, enolase, and heat shock proteins with different expressions between breeds. Another study that compared Belgian Blue and Aberdeen Angus steers observed several proteins with different regulation between breeds that were related with high growth performances [21]. The application of proteomics to compare different breeds can be useful to gain insights into the molecular basis of breed-specific characteristics, such as meat quality, and to improve and increase the efficiency of breeding programs through the identification of proteins related to higher productivity and desirable traits [22].

To the best of our knowledge, in addition to a lack of studies using Omics in Portuguese autochthonous breeds, there is also a gap in the detailed description of why PDO beef products are more nutritious and of higher quality when compared to that of commercial breeds. Moreover, there is a lack of studies comparing standard commercial crossbred with traditionally produced beef. For this reason, with this study, it will be possible to describe how genetics affects the proteome, affecting several production parameters, such as animal growth and, also, meat traits. Furthermore, it will also be an important step in the traceability and authenticity of the PDO products, describing a set of proteins that can characterize the Arouquesa PDO beef. The main goal of this study was to use 2D-DIGE to compare the proteomic profiles of Arouquesa beef and crossbred beef and describe a set of proteins that can distinguish the PDO beef from a commercial beef. Additionally, we assessed several meat chemical and quality parameters that can also define beef’s nutritional value.

## 2. Materials and Methods

### 2.1. Sample Collection and Meat Chemical Composition

Samples were collected from a certified abattoir and kept at −80 °C until further analysis. We collected and analyzed 5 samples of the Longissimus thoracis muscle for each genetic background (5 of Arouquesa and 5 of crossbred). The samples were collected 24 h after slaughter, from the fifth to the sixth thoracic vertebrae of the left half of the carcass, and only from male animals. Arouquesa weaners were produced in the breed’s traditional system as described by Sacarrão-Birrento et al. [4]. Briefly, the calves are kept indoors and suckle only in the morning and in the afternoon since the cows graze during the day. The animals were weaned and slaughtered at 8 months. At three months, animals were fed with hay, green fodder, and small percentages of ground maize. The crossbred weaners were Limousin and Charolais (2 Charolais and 3 Limousin) crossbred with a small percentage of the reverse (Charolais and Limousin). The animals were produced in the highlands of Miranda do Douro (Northeastern Portugal), and calves were kept with their mother on grazing until 3–4 months and then kept indoors, suckling in the morning and in the afternoon. Animals were then fed with concentrate feeding and wheat straw and slaughtered at 8 months of age.

Several meat quality analyses were performed. Meat samples were lyophilized using a CoolSafe Superior Touch 95 freeze dryer (Labogene, Alleroed, Denmark) at 92 °C and 0.2 hPa and ground with a coffee grinder (Stainless Steel Deluxe, Princess, Tilburg, The Netherlands) for energy quantification and fatty acid (FA) analysis. The Kjeldahl method was performed to determine the nitrogen (N) content following the AOAC 954.01 protocol [23]. For the crude protein content calculation, a factor of 6.25 was used, and results were expressed as a percentage of fresh material. The energy content of meat was obtained using an adiabatic bomb calorimeter and results were expressed in cal/g of fresh weight (Parr 6400 1261, Parr Instrument Company, Moline, IL, USA).

Additionally, the pH determination was carried out 24 h after slaughter, directly on the meat with a penetration combined pH electrode coupled to a WTW 330i potentiometer (WTW GmbH, Weilheim, Germany).

### 2.2. Minerals

The mineral profiles of beef were analyzed as described by Ribeiro and co-workers [24]. Briefly, in a digestion tube, 0.3 g of freeze-dried sample was dissolved in 3 mL of concentrated nitric acid and 10 mL of hydrochloric acid for approximately 16 h. Before digestion, 1 mL of hydrogen peroxide was added, and then the tubes were placed in a digestion plate (DigiPREP MS, SCP Science, Quebec, QC, Canada). The tubes were heated for 1 h until the temperature reached 95 °C and then for 1 h at 95 °C. After the digestion, samples were kept in a ventilated chamber to cool. Finally, samples were diluted in a 25 mL volumetric flask with distilled water and then filtered with filter paper of 90 mm (Filter-Lab ref. 1242, FILTROS ANOIA S.A., Barcelona, Spain). Readings by ICP-OES (Inductively coupled plasma–optical emission spectrometry) were performed in an iCAP 7200 ICP-OES spectrometer with an automated sampler (Thermo Scientific, Waltham, MA, USA). The calibration curves were created with multi-element standards (SPC Science, PlasmaQual S22), and multi-element detection and quantification were carried out overnight. The results were expressed in mg/100 g of fresh weight.

### 2.3. Fatty Acid Analysis

For fatty acid analysis, first lipids were extracted from lyophilized muscles using a modified version of the Folch [25] method as described by Alves et al. [26]. The extracted lipids were then *trans*-esterified to produce fatty acid methyl esters (FAME) following a procedure adapted from Alves et al. [26]. The FAME separation was conducted by gas–liquid chromatography with flame ionization detection as described by Alves et al. [26].

### 2.4. Sample Preparation for 2D-DIGE

Fifty milligrams of each meat sample were weighed into reaction vials together with 1 mg of ceramic beads (QIAGEN, Düsseldorf, Germany) with 1.4 mm diameter and 500 μl of buffer (7 M urea, 2 M thiourea, 2% CHAPS, 0.03 M tris-HCl, pH 8.5, proteinase inhibitor (Sigma, St. Louis, MO, USA)). Samples were homogenized using a MagNA Lyser (Roche, Basel, Switzerland) two times for 20 s at 6000 rpm with intermediate cooling. Then, samples were placed in the Thermomixer at room temperature for 1 h at 700 rpm. Finally, samples were centrifuged at 10,000 rpm and the supernatants were collected. The protein concentration was determined using the Bradford protein assay.

### 2.5. 2D-DIGE

2D-DIGE was performed based on described methodologies [18]. Labeling with CyDyes (GE Healthcare, Munich, Germany) was performed with Cy3 and Cy5 for the single meat samples, whereas Cy2 was used as internal standard (labeling ratio: 8 nmol dye/mg protein). Reverse labeling was included for all the samples.

For each gel, extracts composed of a mixture of Cy3- and Cy5-minimally labeled samples and Cy2-labeled internal standard, overall 75 µg, were diluted with rehydration solution (8 M urea, 4% CHAPS, 13 mM DTT, 1% ampholytes). The sample mix was rehydrated into 11 cm long IPG strips with a gradient pH 3–10 (Serva, Heidelberg, Germany) according to manufacturer recommendations, and first-dimensional IPG was performed overnight in a Multiphor II electrophoresis system (GE Healthcare, Uppsala, Sweden). Strip equilibration was followed by the second-dimensional separation using a Hoefer SE600 system (Hoefer Scientific Instruments, San Francisco, CA, USA) on an SDS-PAGE gel (140 ×140 × 1.5 mm home-made gradient gel; T = 10–15%, C = 2.7%). Gel scanning was done in a Typhoon RGB (GE Healthcare, Uppsala, Sweden) and images analyzed by the software DeCyder V6.0 (GE Healthcare, Uppsala, Sweden), selecting the spots differentially regulated by the fold-change (±1.5) and *p*-value (*p* < 0.05). Additionally, gels were stained after scanning with an MS-compatible silver stain, to enable localization of the spots by eye for manual spot-cutting. Staining also revealed the two additionally applied molecular weight markers, LMW molecular weight marker (GE Healthcare, Uppsala, Sweden) and Serva Triple Colour Protein Standard III (Serva, Heidelberg, Germany).

### 2.6. Protein Digestion of 2D Spots

Spots of interest were cut from gels, washed and destained using 30 mM potassium ferricyanide and 100 mM sodium thiosulfate, both dissolved in water as a 1:1 ratio working solution. After one washing step with 100 mM ammonium bicarbonate (NH_4_HCO_3_) and a drying step using acetonitrile and a vacuum concentrator (Eppendorf, Hamburg, Germany), further washing, reduction, and alkylation were performed according to Shevchenko et al. [27]: Dried gel spots were swollen in 10 mM dithiotreitol (DTT) in 100 mM NH_4_HCO_3_, and the disulfide bonds of the proteins reduced for 1 h at 56 °C. DTT was removed and replaced by 55 mM iodoacetamide in 100 mM NH_4_HCO_3_ for alkylation for 45 min at 25 °C in the dark. After two washing steps with 100 mM NH_4_HCO_3_ for 5 min and dehydration using acetonitrile and a vacuum concentrator, gel pieces were rehydrated in digestion buffer containing 50 mM NH_4_HCO_3_, 5 mM calcium chloride, and 12.5 ng/μL of trypsin (Trypsin Gold, Promega, Alexandria, Australia), and proteins were digested for 8 h at 37 °C. Peptides were extracted by three changes of 5% formic acid in 50% acetonitrile (10 min for each change) and dried down in a vacuum concentrator. Peptides were resuspended in 8 µL 0.1% TFA for LC–MS/MS analysis (injection volume 6 µL).

### 2.7. LC–MS/MS Analysis and Protein Identification

Peptides were separated on a nano-HPLC Ultimate 3000 RSLC system (Dionex, Waltham, MA, USA) and detected by a QExactive HF mass spectrometer (Thermo Fisher Scientific, Waltham, MA, USA) according to Mayr et al. [28] using a modified 30 min HPLC gradient with a flow rate of 300 nL/min. It started with 4% mobile phase B (80% ACN with 0.08% formic acid) for 7 min, increased to 31% in 30 min and to 44% in an additional 5 min. It was followed by a washing step with 95% B. Mobile Phase A consisted of ultra-pure H_2_O with 0.1% formic acid.

MS acquisition parameters are described in detail in Mayr et al. [28]. In brief, full MS scans were acquired from m/z 350 to 2000 at a resolution of 60,000, with an automatic gain control (AGC) target of 3 × 10^6^ ions and a maximum injection time of 50 ms. MS^2^ scans of the ten most intense masses were performed at a resolution of 15,000 with the intensity threshold at 4 × 10^4^ and a maximum injection time of 50 ms.

A database search for acquired mass spectra was accomplished in Proteome Discoverer 2.4.1.15 with the Sequest HT search engine (Thermo Fisher Scientific, Waltham, MA, USA) using a Bos taurus database (taxonomy ID 9913, downloaded from www.uniprot.org, accessed on 25 August 2022, 47.140 entries) combined with a common contaminant database (http://www.thegpm.org/crap/, bovine BSA and keratins removed manually, accessed on 25 August 2022). Search parameters were set to 10 ppm precursor mass tolerance and 0.02 Da fragment mass tolerance. Trypsin was set as a digestion enzyme with maximally two missed cleavages tolerated. The dynamic modifications included were oxidation (M), deamidation (N, Q), and Gln to pyro-Glu (Q on peptide terminus). As a static modification, carbamidomethylation (C) was determined. Target decoy analysis was performed by searching a reverse database with a strict false discovery rate (FDR) of 0.01 and a relaxed FDR of 0.05 at the protein and peptide level. Only proteins identified with at least two peptides, with at least one unique peptide and with the highest score (score of second-best hit < 50% of top hit) were taken into account in the evaluation.

### 2.8. Statistical Analysis

For the statistical analysis, the SAS software (version 9.4, SAS Institute Inc., Cary, NC, USA) was used. For all the parameters except for FA, data was analyzed by one-way analysis of variance (ANOVA), using the General Linear Model (GLM) procedure. For FA, data was analyzed using the Mixed procedure (PROC MIX). The genetic background (Arouquesa or crossbreeding) was the single effect. A verification of distribution normality and variance homogeneity through the Shapiro–Wilk test was included as well. The results were considered statistically different with *p*-value < 0.05. Additionally, the *p*-values were adjusted using the Benjamini–Hochberg false discovery rate (FDR) control method. Results with FDR < 0.05 were considered significant.

### 2.9. Functional Analysis

The STRING database version 12.0 [29] was used to conduct a protein–protein interaction network of the differentially abundant proteins between Arouquesa and crossbred animals and to perform an enrichment analysis. A minimum required interaction score of ≥0.7 (high confidence) was chosen with only query proteins for the first shell and no interactions for the second shell. In the functional analysis by the Biological process (Gene Ontology), grouping the terms with a similarity of ≥0.8 and a maximum FDR of 0.05 was selected.

## 3. Results

### 3.1. Meat Quality Parameters

Table 1 presents observed parameters related to the animals as well as the beef chemical and meat quality traits. The final liveweight and the crude protein and energy were statistically different (*p*-value < 0.05) between breeds. The Arouquesa breed showed lower liveweight (132 kg vs. 214 kg) and lower percentage crude protein (17.5%) content, while the gross energy content was higher in the Arouquesa breed.

#### 3.1.1. Mineral Profiles

The mineral concentration of beef is presented in Table 2. No significant differences between breeds were detected concerning all macrominerals, including total minerals. On the other hand, there were statistical differences (*p*-value < 0.05) concerning the total of microminerals, the zinc and manganese content being higher in the Arouquesa breed beef.

#### 3.1.2. Fatty Acid Profile

Table 3 shows the results for the muscle FA composition from Arouquesa and crossbred animals. The total saturated FA (SFA) was the class of highest abundance in both muscles, but Arouquesa showed 15.7% more SFA when compared to crossbreds. The proportions of 16:0 and 18:0 contributed to such differences, as together they counted for 44.3% and 39.7% of total FA, respectively. Although the 16:0 proportion did not show differences between muscles, the proportion of 18:0 was highest (17.9% and 13.9% total FA) in the Arouquesa breed. For the other SFA, the 10:0 and 20:0 proportions were also in higher proportion (*p* < 0.05) in Arouquesa beef.

Regarding the total BCFA, higher proportions were observed in Arouquesa cattle, including the i-14:0, i-15:0, i-17:0, and i-18:0. On the other hand, the *cis*-MUFA tended (*p* = 0.052) to be higher in the crossbred muscle, however only minor isomers differed between meats. From these, the *c*9-17:1 and *c*13-18:1 had higher proportions in the crossbred animals, whereas the *c*15-18:1 was higher in Arouquesa. Among *trans*-MUFA, the most abundant were the *t*10-18:1 and *t*11-18:1, but only *t*11-18:1 differed between breeds, with Arouquesa having the higher proportion (1.09 vs. 0.67% total FA). Moreover, the *t*12-18:1 was higher in crossbred animals, while the *t*16-18:1 was higher in the Arouquesa breed. For the other FA, including omega-3 and omega-6, no significant differences (*p* > 0.05) were found between breeds.

Regarding the ratios with nutritional interest, only the h/H (hypocholesterolemic/hypercholesterolemic) was higher in the crossbred beef compared to the Arouquesa. Also, in crossbred animals, the *t*10-shift tended (*p* = 0.066) to be 2.5 times higher when compared to the Arouquesa beef.

### 3.2. Proteomic Analysis

**Figure 1:**
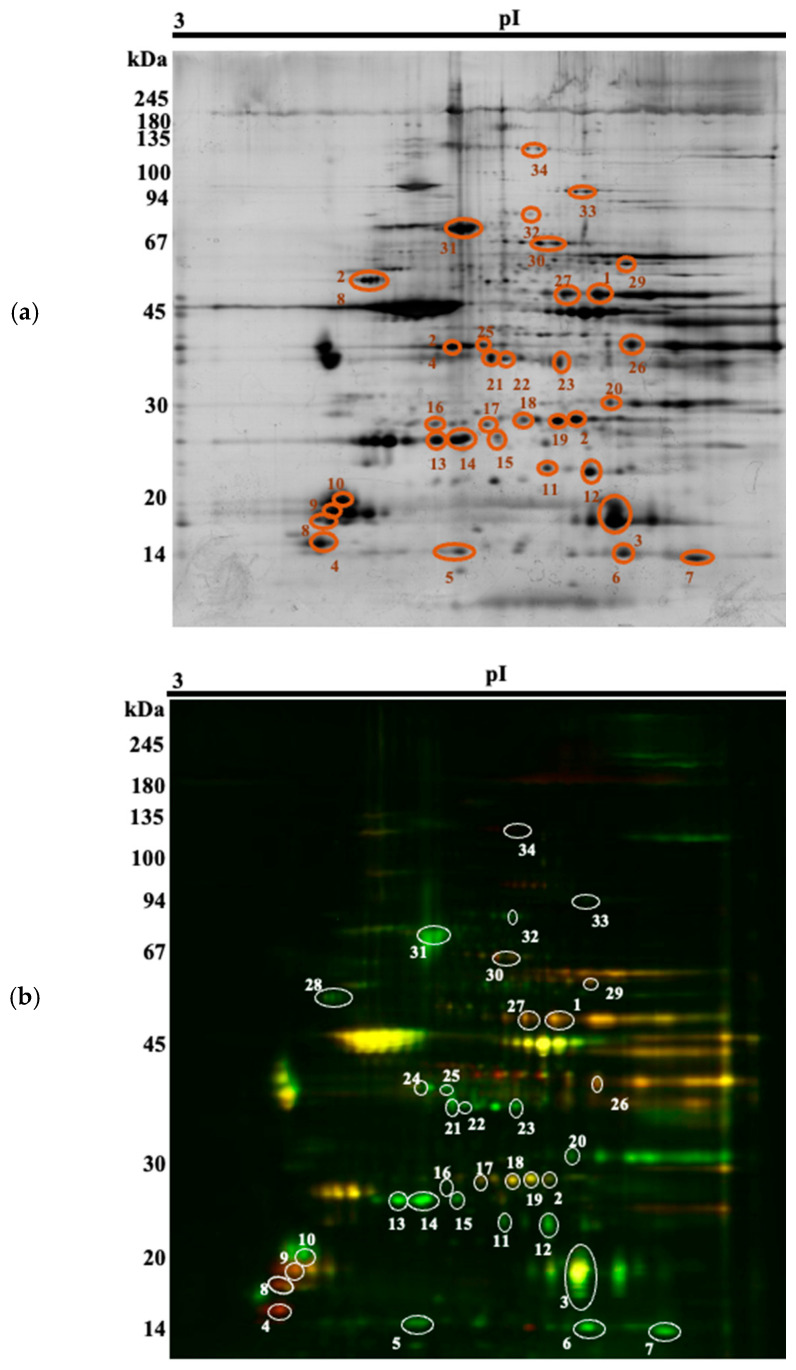
Representative 2-D DIGE gel with the respective 34 spots of interest (numbered 1 to 34). (**a**) Silver stain; (**b**) DIGE image (green corresponding to Arouquesa, red to crossbred, similar spots overlapping to yellow).

Thirty-four spots of interest were selected (Figure 1) for MS-identification (Supplementary Material S1), based on a *p*-value (*p* < 0.05) and a fold-change of at least ± 1.5. They could be attributed to 24 different proteins. Among the spots, 13 were upregulated in the Arouquesa beef, whereas 21 were upregulated in the crossbred beef (Table 4). Some of these differentially abundant spots were identified as the same protein, most of them with different isoelectric points and similar molecular weights, most likely indicating different isoforms or post-translational modifications (ENO3, TPI1, MYLPF, CRYAB, MYL6B, HSPB1, TNNT1). Not all of these proteoforms were regulated in the same way. For instance, spots 1 and 28 were identified as ENO3 and both were downregulated in the Arouquesa breed. On the gels, their pIs corresponded to 7.12 and 7.44, respectively, whereas the theoretical pI for ENO3 is 7.72 (Table 4). On the other hand, TPI1 was downregulated in Arouquesa in spots 18 and 19 (pI of 6.66 and 7, respectively) and in crossbred in spot 2 (pI of 7.16). In addition, MYLPF had a similar pattern. Spots 8 and 9 were downregulated in Arouquesa, while spot 10 was upregulated. Besides different pI values, the last three spots mentioned also differed in molecular weights. Spot 8 had a molecular weight of 17 kDa, spot 9 of 18 kDa, and spot 10 of 19 kDa.

The first main term was muscle contraction (GO:0006936) with myosin light chain 1/3 (MYL1) in higher abundance in crossbreds (spot 4), myosin regulatory light chain 2 (MYLPF) with different spot abundance in Arouquesa (spot 10) and crossbred (spots 8 and 9), and troponin T, slow skeletal muscle (TNNT1) in higher abundance in Arouquesa (spots 22, 24, and 25). Another main term was the glycolytic process (GO:0006096) with beta-enolase (ENO3) in higher abundance in crossbreds (spots 1 and 27), triosephosphate isomerase (TPI1) with different spot abundance in Arouquesa (spot 2) and crossbred (spots 18 and 19). The third main term was the hydrogen peroxide catabolic process (GO:0042744) with globin A1 (HBB), hemoglobin subunit alpha (HBA), both highly abundant in Arouquesa (spots 6 and 7, respectively) and myoglobin (GLNG) highly abundant in crossbred (spot 3).

Other upregulated proteins in Arouquesa animals were the following: serotransferrin (TF) involved in actin filament organization (GO:0007015) in spot 32; myosin light chain 6B (MYL6B) related to calcium ion binding (GO:0005509) in spots 13, 14, and 15; heat shock protein beta-1 (HSPB1) related to chaperone-mediated protein folding (GO:0061077) in spots 16 and 17; fatty acid-binding protein (FABP3) involved in the long-chain fatty acid transport (GO:0015909) in spot 5; and alpha-crystallin B chain (CRYAB) related to the response to heat (GO:0009408) in spots 11 and 12.

Spots with more than one protein identified with a high score were disregarded in the evaluation, although their regulation may also be different between breeds.

**Figure 2:**
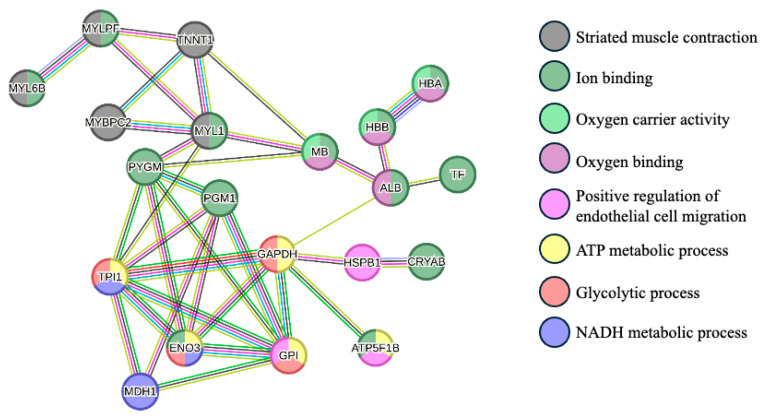
Protein–protein interaction network of the differentially abundant proteins between Arouquesa and crossbred animals. The nodes represent proteins from the Bos taurus database, whereas the edges represent protein–protein associations (light blue—known interactions from curated databases; pink—known interactions experimentally determined; dark green—gene neighborhood evidence; red—gene fusions evidence; blue—gene co-occurrence evidence; light green—text mining; black—co-expression; purple—protein homology). The colors of the nodes represent the biological process in which the proteins are involved (gray—striated muscle contraction; dark green—ion binding; light green—oxygen carrier activity; dark pink—oxygen binding; light pink—positive regulation of endothelial cell migration; yellow—ATP metabolic process; red—glycolytic process; purple—NADH metabolic process. MYL6B—Myosin light chain 6B; MYLPF—Myosin regulatory light chain 2; TNNT1—Troponin T; MYBPC2—Myosin binding protein C2; MYL1—Myosin light chain 1/3; MB—Myoglobin; ALB—Albumin; HBB—Globin A1; HBA—Hemoglobin subunit alpha; TF—Serotransferrin; PYGM—Alpha-1,4 glucan phosphorylase; PGM1—Phosphoglucomutase-1; TPI1—Triosephosphate isomerase; GADPH—Glyceraldehyde-3-phosphate dehydrogenase; HSPB1—Heat shock protein beta-1; CRYAB—Alpha-crystallin B chain; ENO3—Beta-enolase; MDH1—Malate dehydrogenase, cytoplasmic; GPI—Glucose-6-phosphate isomerase; ATP5F1B—ATP synthase subunit beta. This network was carried out using STRING software version 12.0 [29].

The protein–protein interactions are represented in Figure 2. This analysis showed mainly three clusters between proteins: proteins that are related to the striated muscle contraction (MYL6B, MYLPF, TNNT1, MYBPC2, and MYL1), proteins with oxygen carrier activity and oxygen binding (HBA, HBB, MB, and ALB), and proteins involved in glycolytic and ATP metabolic processes (TPI1, GAPDH, ENO3, and GPI).

**Figure 3:**
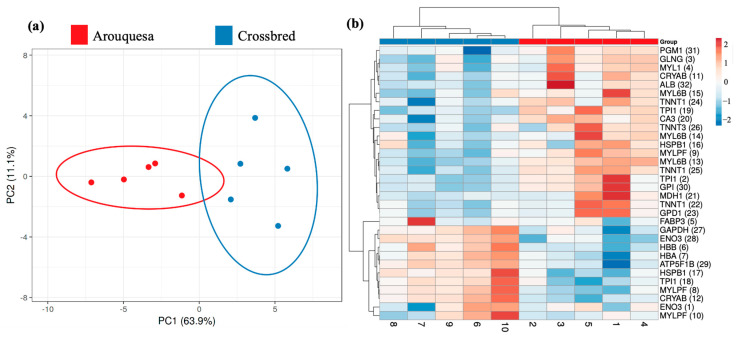
(**a**) Principal component analysis (PCA) for the differential spots among Arouquesa and crossbred muscle. (**b**) Heatmap view of the identified differential proteins. The horizontal tree represents the samples from each genetics (Arouquesa and crossbred). The vertical tree represents the identified proteins with the corresponding number of spots in parentheses. The color indicates the relative volume for each spot.

Figure 3 shows the principal component analysis (PCA) and heatmap comparing the groups in study. The PCA shows a clear separation between groups and the PC1 explains 63.9% of the variability. Also, in the heatmap, it is possible to distinguish the groups.

## 4. Discussion

The present study is the first approach to compare the proteome profiles of the Arouquesa breed produced under the traditional system of the PDO beef with crossbred beef produced under standard conditions.

At the same age, the crossbred animals were expected to have higher liveweight than the Arouquesa breed, as demonstrated in other studies that compared local breeds with genetically improved breeds [30,31,32]. In addition, crossbred beef had higher crude protein contents than Arouquesa beef. A previous study [33] compared the muscle from Limousin bulls fed with different diets and showed a higher proportion of protein content in the meat of animals fed with a higher percentage of concentrate. Another study, that compared Limousin bulls with a local breed from Poland (Polish Red), found a higher protein content in the first breed (24.2%) when compared to the latter (21.7%), explaining this value by the genetic influence [34]. In our study, both feed and genetics were different, which led to higher crude protein values in the crossbred beef. The fact that the crossbred animals had a higher final liveweight than the Arouquesa animals resulted in a higher body mass at slaughter and, consequently, higher protein content in muscle [35]. On the other hand, Arouquesa animals had a higher value of crude energy. The recommendations for caloric intake for human health are dependent on several factors, such as gender, age, hormonal status, dieting behaviors, pregnancy, etc. [36]. This value could be affected by the possible difference between the muscle fibers from the different breeds, but also by the feeding system. The fact that these two genetics are very different, could have led to different transformations in the muscle fiber leading to the Arouquesa breed having a higher glycogen content in the post-mortem [37]. The glycogen content and glycolytic potential contribute to higher levels of available energy substrates, leading to higher gross energy values. The different feeds could also have affected the fat deposition, leading to this difference. These results have nonetheless to be confirmed with further studies on muscle histology.

Concerning the mineral profile, Arouquesa beef had higher levels of micronutrients including Fe, Zn, and Mn. According to García-Vaquero et al. [38], when animals have a balanced mineral status, the essential trace element content is affected by their own metabolism. There was a study that compared Limousin, Angus, Salers and others with Hereford, Limousin, and Charolais that showed an influence of the genotype in the trace mineral content [39,40]. In the present study, the Fe, Zn, and Mn content in Arouquesa breed was 2.04, 4.73, and 0.02 mg/100 g of meat, respectively. According to the dietary guidelines for humans [41], the recommended daily values are 8–18, 8–11, and 1.6–2.3 mg, respectively. Because the Arouquesa breed had higher values for certain minerals, it can be considered more nutritious, and the amount of consumption can be lower to satisfy the needs of these elements in humans.

Regarding the FA profile, the genetic group affected the SFA content, including the 10:0, 18:0, and 20:0 proportions. This difference can be a result of the preferential deposition of SFA and MUFA, especially the *c*9-18:1, in the triacylglycerol fraction that is positively correlated with the intramuscular fat content [42]. Nevertheless, neither the *cis*-MUFA nor the *c*9-18:1 was affected by the genotype, and the FA that most contributed to the high proportion of SFA in Arouquesa samples was 18:0, which is known to be the end product of rumen biohydrogenation of dietary unsaturated FA [43]. It can be postulated that the biohydrogenation of dietary C18 PUFA in the rumen was highest for the Arouquesa breed, or a lower activity of delta-9 desaturase enzyme was responsible for the conversion of 18:0 into *c*9-18:1 in tissues, resulting in a higher deposition of 18:0 in intramuscular fat. From a nutritional point of view, 18:0 is an SFA, but less harmful to human health than 16:0 or industrial *trans*-FA [44,45].

The *trans* fatty acids found in muscle fat are considered biohydrogenation intermediates formed in the rumen during the microbial biohydrogenation process of dietary PUFA. Some of these biohydrogenation intermediates can then escape the rumen, such as the *t*11-18:1 that is formed in the rumen and the *c*9,*t*11-18:2 in tissues, a conjugated linoleic acid (CLA) known for its positive biological effects [46]. In our study, no differences were observed in CLA between genotypes, but the *t*11-18:1 was higher in Arouquesa intramuscular fat. Despite that, the major *trans*-fatty acid in muscle was the *t*10-18:1, averaging 1.4% of total FA. Higher proportions of *t*10-18:1 compared with *t*11-18:1 are referred to as *trans*-10 shift [47] and represent a change in the normal biohydrogenation pathways where the *t*11-18:1 is replaced by *t*10-18:1 as the main *trans*-MUFA formed in the rumen. The *t*11-18:1 has positive biological effects on human health, while the biological effects of *t*10-18:1 are associated with various adverse health outcomes, including an increased risk of heart disease [48]. The formation of *t*10-18:1 in the rumen and its deposition in tissues is associated with ruminants being fed low-fiber, high-starch diets, and thus being associated with concentrate feeding. So, because the *t*10-shift (ratio *t*10-18:1/*t*11-18:1) was particularly higher than 1 in crossbred samples, we can postulate that the dietary forage to concentrate ratio was lower in these animals compared to the Arouquesa breed.

Some of the BCFA (i-14:0, i-15:0, i-17:0, i-18:0) were higher in the Arouquesa beef, possibly indicating differences in the microbial population in the rumen. Indeed, these FA are of microbial origin in the rumen, and the levels of starch and fiber in the diet can influence the microbiota and, therefore, the FA from microbial membranes that can escape from the rumen, being absorbed in the small intestine and deposited in the tissues [43]. Although we do not have information about the composition of the feed, these results lead us to suggest again that the crossbred had a higher consumption of concentrate with high proportions of starch, which is negatively correlated with some of these BCFA [49]. Additionally, as referenced by another study conducted on the Arouquesa breed [50], the fact that the milk has high proportions of BCFA, and this breed being dependent on the mothers’ milk in the traditional system, can also be a justification.

Although there was a considerable numerical difference between the genotypes on total n-6 PUFA (2.93 vs. 6.49 % total FA) and n-3 PUFA (0.71 vs. 0.96% total FA), no statistical differences were observed. This lack of statistical significance may be attributed to the high variability and small sample size in each group. Similarly, the n-6/n-3 ratio was numerically higher in the crossbred beef, but in both groups, it was higher than 4.0. A very high n-6 and n-3 PUFA ratio is very important for human health as it is linked to many diseases, including cardiovascular disease, cancer, and inflammatory conditions. Conversely, a lower ratio has been associated with protective effects [51]. Thus, it is recommended not to exceed 4.0 [52]. However, Arouquesa beef presented a value of 4.83, and the crossbred had a value of 6.98. Nevertheless, none of the groups had extremely high values that were reached in other studies with concentrate-fed animals (ratio n-6/n-3 between 13 and15) [53,54]. The ratio h/H was higher in the crossbred beef. This ratio is considered by some authors to be a more reliable indicator of the nutritional value of meat lipids as it characterizes the functional effects of the fatty acids, mainly on the cholesterol metabolism [55]. The higher value found in crossbred beef points out to healthier meat in terms of cholesterolemic effects.

Concerning proteomics, the proteome patterns of glycolysis and gluconeogenesis related proteins (TPI1 and GPD1) show several differences. For TPI1, the two more acidic spots of this protein were increased in crossbreds, whereas the alkaline spot was more abundant in Arouquesa. A shift to more acidic spots could be the sign for differences in PTMs (e.g., phosphorylation), which requires further confirmation with other measures. Glycolysis after slaughter is a key factor in the post-mortem period since it determines the glycogen content. The amount of glycogen plays a crucial role in determining the ultimate pH of the meat. This, in turn, affects numerous meat characteristics, including color, tenderness, and water losses [56]. TPI1 is involved in the interconversion of dihydroxyacetone phosphate and glyceraldehyde-3-phosphate and in the biosynthesis of triacylglycerol and FA, being most likely related to the accumulation of intramuscular fat (IMF) [57]. In fact, some studies with a Korean cattle breed (Hanwoo) showed that TPI1 is a potential biomarker of animals with higher levels of IMF [57,58]. On the other hand, being part of sarcoplasmic protein fraction and their denaturation having a role in water losses, this protein can also be a potential biomarker of several meat quality aspects, such as water holding capacity, drip losses, and pH [59]. In addition, in a study comparing Nellore and Angus, TPI1 phosphorylation was associated with tenderness [60]. Additionally, GPD1 was also related to IMF in a study that compared different diets in Angus-Nellore bulls [61]. In our study, we did not report differences in meat quality parameters between Arouquesa and crossbred animals. Nonetheless, we can hypothesize that there were differences in the muscle post-mortem mechanisms, including not only the glycogen degradation between breeds affected by the genetics but also by the differences in the production system.

The Arouquesa beef had a higher abundance of proteins related to muscle contraction (TNNT1 and one of the three proteoforms of MYLPF) and calcium binding (MYL6B). Myosins are found in thick filaments, and their degradation occurs in the early post-mortem period having an important role in beef tenderness [62]. For this reason, it is expected that Arouquesa beef will have higher tenderness (albeit not measured here). MYLPF was identified in three spots with opposite regulation patterns, suggesting different isoforms or post-translational modifications, although the low number of analyzed samples can also influence these results. Mato et al. [63] reported that highly phosphorylated MYLPF isoforms showed different regulations between normal and dark, firm and dry (DFD) meat. This illustrated that phosphorylation could vary based on meat characteristics such as color, water holding capacity, and pH, traits that are often variable between breeds. In our study, the fact that two spots are downregulated in Arouquesa and one in crossbred beef indicates breed-specific different proteoforms of MYLPF that may be due to differences in meat quality traits. However, it is important to measure more quality parameters in the future to confirm these results. TNNT1 is involved in the muscle contraction of slow-twitch muscle fibers, so the abundance is affected by the type of muscle [64]. This protein was positively correlated with some meat quality traits in a study with PDO Maine-Anjou cows (a representative of Rouge de Prés French breed) as IMF and tenderness [65]. Although in our study the animals were slaughtered at the same age the presence of TNNT1 may point to differences in muscle fibers, due to the very different animals’ genetics, together with the production system differences. Additionally, as previously mentioned, the likely difference in tenderness can be explained by the presence of this structural protein.

Other proteins that concur to a possible difference in the type of muscle are the oxygen transport proteins (HBA and HBB). At post-mortem, the levels of hemoglobin are very low in the muscle, thus, the presence of these proteins can be due to differences in the muscle fibers [66]. The amount of hemoglobin is related to the oxidative status of muscle and the oxidation of hemoglobin and myoglobin affects the meat color in giving an undesirable appearance to the consumers [67]. So, the higher abundance of these proteins, as indicated in the gel pattern, points to a darker meat in Arouquesa beef. These results require further analysis on beef color.

In the current study, heat shock protein beta-1 (HSPB1), which is related to cell defense and development and signal transduction [21], was upregulated in Arouquesa beef. HSPB1 is a key regulator of actin polymerization, being essential to muscle structure and contributing to muscle development through the inhibition of protein degradation [68]. Together with CRYAB (also upregulated in Arouquesa), this protein can attach to myofibrils, forming protein complexes and protecting the skeletal muscle [69]. The protective effect has a negative correlation with tenderness and was also associated with beef color [70]. However, in our work we did not study these parameters. The fact that these proteins are related to stress leads us to hypothesize that due to genetic background and differences in the production environment, responses to stress could be different.

Several proteins upregulated in crossbred beef were involved in glycolysis/gluconeogenesis (PGM1, ENO3, GADPH, GPI, two proteoforms of TPI1). Limousin and Charolais are known for high muscle growth, with low proportions of fat and high proportions of fast glycolytic fibers, as described [71]. These types of fibers predominantly use glycolytic pathways to produce energy. This affects several meat quality traits such as pH, color, and tenderness [72], which can explain the higher presence of the glycolytic proteins in the crossbred beef. Among these proteins, PGM1 was related with tenderness in several studies [73,74], because it affects the balance between G-1-P and G-6-P and can have several post-translational modifications improving the glycolytic potential and consequently the rate of pH decline [75]. Furthermore, glucose metabolism is a crucial metabolic pathway that provides carbon and reduces necessary cofactors for intramuscular fat [60]. In fact, Suh et al. [68] compared two breeds with different muscle growth rates (crossbred Aberdeen Angus and Belgian Blue) and showed that the presence of proteins related to glycolysis can indicate differences in the IMF. However, another study found a relationship between these proteins and bulls selected for high muscle growth [76]. An additional glycolytic protein that showed higher abundance in the crossbreds was ENO3. This protein has been linked to meat quality traits, particularly with a positive correlation to tenderness [77]. In a study that analyzed the proteomic profile of animals with different age and sex, ENO3 was abundant in younger animals that, when compared to bulls, have more glycolytic muscle [78]. Once again, this result points to a difference in the muscle fiber composition between Arouquesa and crossbreds, that has to be confirmed in future studies. ENO3 was identified in two spots with different pI, pointing to post-translational modifications, but both were lower in Arouquesa. ENO3 catalyzes the conversion of 2-phospho-D-glycerate to phosphoenolpyruvate, and phosphorylation of the enzyme beta-enolase increases the phosphoenolpyruvate synthesis [79]. According to Mato et al. [63], ENO3 phosphorylation is observed in dark meats and can be a response to meet the increased energy requirements after pre-slaughter stress. In our study, it can also represent a response to stress since the slaughter age (8 months) was lower than usual and younger animals are more susceptible to stress [4].

On the other hand, the crossbred beef had proteins related to muscle structure development and contraction (MYL1, 2 slightly smaller proteoforms of MYLPF). The proteins involved in muscle development are associated with higher growth rates and consequently higher muscle deposition, as described in other studies [80,81,82]. In our study, the crossbred animals had higher final liveweights pointing to higher muscle development, a fact that can explain the higher abundance of these proteins.

## 5. Conclusions

This study is a first approach to compare the Arouquesa breed with commercial beef, describing a set of proteins that in the future can be validated and used in PDO certification. The genetics of these animals are different, as well as the production system, so when they were slaughtered at a young age (8 months), some differences were to be expected. We observed differences in the liveweight, which was foreseen because we were comparing a local breed with low production rates with breeds that were selected in the last years for high growth rates. Consequently, we found differences in the protein, energy, and fatty acid profile of muscle. Additionally, the Arouquesa breed had a higher content of some microminerals that can be important for human health. By using proteomics, it was possible to find altered protein patterns or concentrations for the two animal groups; this is of major significance for the certification process. While Arouquesa beef had upregulated proteins that can reflect higher tenderness, crossbred beef had a higher abundance of proteins that reflect the higher growth rates and which point to differences in muscle fiber composition. However, the limited number of samples and the fact that the samples were collected from an abattoir with limited information on the animals being available, represent several limitations in this study. Furthermore, the variability between samples due to the uncontrolled conditions and the low number of replicates could have had some influence. In the future, it will be important to assess certain aspects of meat quality and muscle composition in more detail, which are probably the main factors that affected some of the results. Additionally, the biomarker validation of proteomics results will be important to contribute to the compliance of certification specifications.

## Figures and Tables

**Table 1:** Meat parameters from the different genetics (Arouquesa and crossbred beef).

	Arouquesa	Crossbred	SEM ^1^	*p*-Value
Liveweight (kg)	132.00	214.00	5.470	<0.001
pH	5.70	5.70	0.050	0.761
Dry Matter (%)	29.63	26.19	1.103	0.058
Crude protein Content (%)	17.52	20.46	0.708	0.019
Gross Energy Content (kcal/100 g)	186.12	143.69	98.585	0.016

^1^ Standard error of mean.

**Table 2:** Mineral content in beef from Arouquesa and crossbred animals.

	Arouquesa	Crossbred	SEM ^1^	*p*-Value
Macrominerals (mg/100 g)				
Sodium	54.74	43.92	3.976	0.070
Potassium	350.53	371.29	11.466	0.217
Calcium	13.42	9.39	2.015	0.186
Magnesium	19.68	20.46	0.663	0.413
Phosphorous	238.86	245.00	8.682	0.623
Sulfur	196.57	197.54	6.184	0.577
Total	868.46	887.60	30.262	0.660
Microminerals (mg/100 g)				
Copper	0.19	0.16	0.013	0.104
Zinc	4.73	2.80	0.148	<0.001
Iron	2.04	1.42	0.097	<0.001
Manganese	0.02	0.01	0.001	0.002
Total	6.98	4.39	0.227	<0.001
Total macro- and microminerals	875.44	892.00	30.382	0.704

^1^ Standard error of mean.

**Table 3:** FA composition (g/100g of total FA) and FA ratios of the muscle from the Arouquesa and crossbred animals. All values are presented as mean ± standard error of the mean.

	Arouquesa	Crossbred	*p*-Value	False Discovery Rate
FA profile				
SFA ^1^	52.74 ± 1.224	45.56 ± 1.224	0.003	0.051
10:0	0.05 ± 0.002	0.04 ± 0.002	0.001	0.051
12:0	0.15 ± 0.023	0.09 ± 0.023	0.133	0.216
14:0	4.60 ± 0.500	3.10 ± 0.500	0.067	0.162
15:0	0.58 ± 0.011	0.46 ± 0.052	0.054	0.162
16:0	26.45 ± 0.674	25.66 ± 0.674	0.432	0.461
17:0	1.11 ± 0.086	0.90 ± 0.086	0.118	0.205
18:0	17.88 ± 0.663	13.90 ± 0.663	0.003	0.051
20:0	0.12 ± 0.008	0.09 ± 0.008	0.011	0.064
22:0	0.01 ± 0.001	0.01 ± 0.001	0.363	0.406
BCFA ^2^	1.79 ± 0.038	1.31 ± 0.147	0.014	0.064
i-14:0	0.06 ± 0.007	0.04 ± 0.007	0.037	0.133
i-15:0	0.20 ± 0.016	0.11 ± 0.016	0.005	0.051
i-16:0	0.21 ± 0.005	0.16 ± 0.023	0.058	0.162
i-17:0	0.44 ± 0.023	0.32 ± 0.023	0.005	0.051
i-18:0	0.13 ± 0.010	0.09 ± 0.010	0.014	0.064
a-15:0	0.23 ± 0.022	0.16 ± 0.022	0.067	0.162
a-17:0	0.52 ± 0.032	0.43 ± 0.032	0.102	0.201
MUFA ^3^				
*cis*-MUFA ^4^	38.62 ± 1.084	42.10 ± 1.084	0.052	0.162
*c*9-14:1	0.52 ± 0.079	0.72 ± 0.079	0.125	0.207
*c*7-16:1	0.31 ± 0.023	0.26 ± 0.023	0.179	0.245
*c*9-16:1	2.48 ± 0.234	3.05 ± 0.234	0.121	0.205
*c*9-17:1	0.52 ± 0.035	0.68 ± 0.035	0.012	0.064
*c*9-18:1	32.75 ± 1.102	34.80 ± 1.102	0.224	0.282
*c*11-18:1	1.09 ± 0.124	1.31 ± 0.124	0.237	0.293
*c*12-18:1	0.18 ± 0.026	0.15 ± 0.026	0.364	0.406
*c*13-18:1	0.17 ± 0.003	0.24 ± 0.018	0.007	0.051
*c*15-18:1	0.12 ± 0.013	0.06 ± 0.013	0.013	0.064
*c*9-19:1	0.11 ± 0.012	0.08 ± 0.012	0.097	0.201
*c*11-19:1	0.05 ± 0.004	0.05 ± 0.004	0.842	0.868
*c*11-20:1	0.11 ± 0.012	0.08 ± 0.012	0.100	0.201
*trans*-MUFA ^5^	3.93 ± 0.320	4.01 ± 0.320	0.867	0.881
*t*6-/*t*7-/*t*8-18:1	0.72 ± 0.018	0.66 ± 0.018	0.064	0.162
*t*9-18:1	0.43 ± 0.026	0.50 ± 0.026	0.109	0.201
*t*10-18:1	1.12 ± 0.283	1.59 ± 0.283	0.278	0.326
*t*11-18:1	1.09 ± 0.085	0.67 ± 0.085	0.008	0.055
*t*12-18:1	0.25 ± 0.060	0.44 ± 0.060	0.057	0.162
*t*16-18:1 ^6^	0.32 ± 0.033	0.15 ± 0.033	0.007	0.051
Non-conjugated dienes				
*c*9,*t*12-18:1 ^7^	0.21 ± 0.024	0.63 ± 0.256	0.179	0.219
*t*11,*c*15-18:2 ^8^	0.14 ± 0.032	0.06 ± 0.032	0.107	0.201
*c*9,*t*12-18:1 ^9^	0.21 ± 0.024	0.63 ± 0.256	0.144	0.219
*c*9,*t*13-/*c*9,*t*14-18:2	0.24 ± 0.031	0.21 ± 0.031	0.566	0.593
*t*8,*c*13-/*c*9,*t*15-18:2	0.14 ± 0.016	0.12 ± 0.016	0.370	0.406
*t*9,*c*12-18:2	0.04 ± 0.002	0.05 ± 0.013	0.903	0.903
Conjugated dienes				
*c*9,*t*11-18:2	0.13 ± 0.018	0.09 ± 0.018	0.155	0.224
*t,t*-CLA	0.31 ± 0.029	0.25 ± 0.029	0.180	0.245
PUFA	4.66 ± 1.593	8.29 ± 1.593	0.146	0.219
n-6 PUFA ^10^	2.93 ± 1.394	6.49 ± 1.394	0.109	0.201
18:2n-6	2.44 ± 1.053	5.08 ± 1.053	0.114	0.204
20:2n-6	0.03 ± 0.009	0.05 ± 0.009	0.142	0.219
20:3n-6	0.10 ± 0.061	0.28 ± 0.061	0.061	0.162
20:4n-6	0.33 ± 0.079	0.97 ± 0.344	0.108	0.201
22:4n-6	0.03 ± 0.024	0.10 ± 0.024	0.076	0.171
n-3 PUFA ^11^	0.71 ± 0.218	0.96 ± 0.218	0.434	0.461
18:3n-3	0.52 ± 0.062	0.42 ± 0.062	0.295	0.340
20:5n-3 (EPA)	0.07 ± 0.015	0.17 ± 0.079	0.280	0.306
22:5n-3 (DPA)	0.11 ± 0.009	0.31 ± 0.138	0.233	0.261
22:6n-3 (DHA)	0.02 ± 0.003	0.07 ± 0.042	0.248	0.276
Ratios				
h/H ^12^	1.17 ± 0.077	1.47 ± 0.077	0.024	0.092
n-6 PUFA/n-3 PUFA ^13^	4.83 ± 1.269	6.98 ± 1.269	0.266	0.318
*t*10-shift ^14^	1.07 ± 0.531	2.67 ± 0.531	0.066	0.162

^1^ Saturated fatty acids. ^2^ Branched-chain fatty acids. ^3^ Monounsaturated fatty acids. ^4^ *cis*-monounsaturated fatty acids. ^5^ *trans*-monounsaturated fatty acids. ^6^ Coelutes with *c*14-18:1 as minor isomer. ^7^ Coelutes with *c*16-18:1 as minor isomer. ^8^ Coelutes with *t*10,*c*15-18:2. ^9^ Coelutes with *c*16-18:1. ^10^ Polyunsaturated fatty acids n-6. ^11^ Polyunsaturated fatty acids n-3. ^12^ h/H (hypocholesterolemic/hypercholesterolemic) = (*c*9–18:1 + 18:2n-6 + 18:3n-3 + 20:4n-6 + 20:5n-3 + 22:5n-3 + 22:6n-3)/(14:0 + 16:0). ^13^ n-6 PUFA/n-3 PUFA = (18:2n-6 + 20:2n-6 + 20:3n-6 + 20:4n-6 + 22:4n-6)/(18:3n-3 + 20:5n-3 + 22:5n-3 + 22:6n-3). ^14^ Ratio between the proportions of *t*10-18:1 and *t*11-18:1.

**Table 4:** Protein identification and average ratio between Arouquesa (A) and crossbred (C) beef. Green spots are upregulated in Arouquesa beef, whereas orange spots are upregulated in crossbred beef.

Spot #	Accession	Description	Gene	pI	Theoretical MW (kDa)	Coverage [%]	Score	Average Ratio C/A
1	Q3ZC09	Beta-enolase	ENO3	7.72	47.1	91	1108.33	1.85
2	Q5E956	Triosephosphate isomerase	TPI1	6.92	26.7	93	900.86	−1.7
3	A0A1K0FUF3	Myoglobin	GLNG	7.46	17.1	100	2291.81	1.36
4	A0JNJ5	Myosin light chain 1/3, skeletal muscle isoform	MYL1	5.02	20.9	67	356.4	3.31
5	P10790	Fatty acid-binding protein, heart	FABP3	7.34	14.8	65	88.13	−2.88
6	D4QBB4	Globin A1	HBB	7.59	15.9	97	339.45	−3.95
7	P01966	Hemoglobin subunit alpha	HBA	8.44	15.2	88	356.77	−2.82
8	Q0P571	Myosin regulatory light chain 2, skeletal muscle isoform	MYLPF	5.01	19	95	255.85	3.07
9	Q0P571	Myosin regulatory light chain 2, skeletal muscle isoform	MYLPF	5.01	19	91	202.02	2.58
10	Q0P571	Myosin regulatory light chain 2, skeletal muscle isoform	MYLPF	5.01	19	93	616.68	−2.17
11	A0A452DHT5	Alpha-crystallin B chain	CRYAB	9.14	25	70	143.49	−1.66
12	A0A452DHT5	Alpha-crystallin B chain	CRYAB	9.14	25	74	526.63	−2.75
13	Q148H2	Myosin light chain 6B	MYL6B	5.53	23.4	92	436.52	−2.68
14	Q148H2	Myosin light chain 6B	MYL6B	5.53	23.4	92	534.56	−3.01
15	Q148H2	Myosin light chain 6B	MYL6B	5.53	23.4	81	112.05	−2.05
16	E9RHW1	Heat shock protein beta-1	HSPB1	6.4	22.4	93	190.77	−1.73
17	E9RHW1	Heat shock protein beta-1	HSPB1	6.4	22.4	95	188.9	−2.28
18	Q5E956	Triosephosphate isomerase	TPI1	6.92	26.7	91	248.96	1.42
19	Q5E956	Triosephosphate isomerase	TPI1	6.92	26.7	95	787.53	1.49
20	Q3SZX4	Carbonic anhydrase 3	CA3	7.84	29.4	83	241.44	−1.44
	E1BKT9	Desmoplakin	DSP	6.84	332.2	23	137.64	
21	Q3T145	Malate dehydrogenase, cytoplasmic	MDH1	6.58	36.4	83	166.46	−2.03
	A0A3Q1M430	Troponin T, slow skeletal muscle	TNNT1	9.79	31.1	42	124.52	
	A0A3Q1M5R4	L-lactate dehydrogenase	LDHB	6.25	37.4	69	124.09	
	Q5KR49	Tropomyosin alpha-1 chain	TPM1	4.74	32.7	70	115.97	
22	A0A3Q1M430	Troponin T, slow skeletal muscle	TNNT1	9.79	31.1	50	439.87	−2.84
23	Q5EA88	Glycerol-3-phosphate dehydrogenase [NAD(+)], cytoplasmic	GPD1	6.89	37.6	96	502.18	−1.32
24	A0A3Q1M430	Troponin T, slow skeletal muscle	TNNT1	9.79	31.1	46	416.55	−2.32
25	A0A3Q1M430	Troponin T, slow skeletal muscle	TNNT1	9.79	31.1	50	305.24	−2.17
26	P10096	Glyceraldehyde-3-phosphate dehydrogenase	GAPDH	8.35	35.8	88	519.32	2.15
	A0A452DJI6	Troponin T, fast skeletal muscle	TNNT3	9.32	45.3	27	331.16	
27	A0A452DI31	Beta-enolase	ENO3	7.72	48.3	66	551.02	1.85
28	P00829	ATP synthase subunit beta, mitochondrial	ATP5F1B	5.27	56.2	84	742.24	−1.58
29	Q3ZBD7	Glucose-6-phosphate isomerase	GPI	7.71	62.8	68	372.91	1.75
30	Q08DP0	Phosphoglucomutase-1	PGM1	6.81	61.6	90	690.56	2.13
31	A0A140T897	Albumin	ALB	6.18	69.3	91	1563.14	−2.22
32	G3X6N3	Serotransferrin	TF	7.17	77.7	78	187.6	−2.21
33	B0JYK6	Alpha-1,4 glucan phosphorylase	PYGM	7.14	97.2	80	694.87	1.82
34	A0A3Q1LQC6	Myosin binding protein C2	MYBPC2	6.79	130.1	60	198.39	2.21

## Data Availability

The original contributions presented in this study are included in the article/Supplementary Materials. Further inquiries can be directed to the corresponding author.

## Supplementary Materials

The following supporting information can be downloaded at https://www.mdpi.com/article/10.3390/ani16010005/s1, Supplementary Material S1: Protein identification.
